# Correlations between risk factors and functional evolution in patients with spastic quadriplegia

**Published:** 2016

**Authors:** OC Rogoveanu, NC Tuțescu, D Kamal, DO Alexandru, C Kamal, L Streba, MR Trăistaru

**Affiliations:** * Department of Physical Medicine and Rehabilitation, University of Medicine and Pharmacy of Craiova, Romania; ** Faculty of Nursing and Midwives, University of Medicine and Pharmacy of Craiova, Romania; *** Department of Medical Informatics and Biostatistics, University of Medicine and Pharmacy of Craiova, Romania; **** Department of Family Medicine, University of Medicine and Pharmacy of Craiova, Romania; ***** Department of Internal Medicine, University of Medicine and Pharmacy of Craiova, Romania

**Keywords:** spastic quadriplegia, risk factors, GMFCS, MACS

## Abstract

Cerebral palsy is the most common cause of developing neuro-motor disability in children, in many cases, the triggering cause remaining unknown. Quadriplegia is the most severe spastic cerebral palsy, characterized by severe mental retardation and bi-pyramidal syndrome. The purpose of this paper was to demonstrate the importance of knowing the risk factors and the psychosomatic ones, determining to what extent they influence the functional evolution in patients diagnosed with spastic quadriplegia. 23 children diagnosed with spastic quadriplegia were included in the study, being aged between 1 year and half and 12 years. Patients were assessed at baseline (T1), at one year (T2) and after two years at the end of the study (T3). Patients received a comprehensive rehabilitation program for the motor and sensory deficits throughout the study. Initially, a comprehensive evaluation (etiopathogenic, clinical and functional) that started from a thorough medical history of children (the older ones), was conducted but chose parents to identify the risk factors, and a complete physical exam. At each assessment, joint and muscle balance was conducted. To assess functionality, the gross motor function classification systems (GMFCS) and manual ability (MACS) were used. Many risk factors that were classified according to the timeline in prenatal factors, perinatal and postnatal, were identified from a thorough history. A direct correlation was noticed between the decrease of coarse functionality and manual ability, both initially and in dynamic and low APGAR scores, low gestational age, low birth weight and a higher body mass index of the mother. A direct link was observed between the gross motor function and the manual ability. A significant improvement in the MACS score was noticed in patients with a better GMFCS score.

## Introduction

Cerebral palsy is one of the most important chapters of infantile neurological pathology, not only in terms of medical therapy and in terms of recovery, but also socially [**1**,**2**]. The spastic quadriplegia form of cerebral palsy means that all the limbs are affected equally, especially the upper limbs, representing 5% of all forms of cerebral palsy. In patients with spastic quadriplegia, the disease affects both the gross motor functions and the fineness, to varying degrees [**2**]. The result is visible both regarding the neuro-motor development, particularly voluntary mobility, and at the level of the motor learning processes. Delayed or inadequate motor developed affects the child’s ability to explore and learn concepts related to the surrounding area, with multiple consequences, motor and social, the behavior and independence [**1**,**3**].

For therapeutic activity-remedial, a special importance is attributed to early diagnosis, enabling the rapid commencement of treatment, thus avoiding the formation of incorrect motor schemes and subsequent fabrication of proprioceptive impulses. A quick diagnosis and a correct knowledge of risk factors are particularly important. The precocity of therapeutic intervention is crucial in achieving an optimal result [**4**,**5**].

The main risk factors that lead to the appearance of spastic quadriplegia are represented by prenatal, perinatal, and postnatal risk factors. Prenatal factors (primary) are responsible for the majority of cases of spastic paralysis, genetic factors playing a particular role between these. Perinatal factors (intrapartum) act from the onset of labor until the delivery. Postnatal causes (postpartum) appear either immediately after birth or late as an infant and toddler, before the nervous system reaches maturity [**1**,**2**].

The purpose of this paper was to demonstrate the importance of understanding the risk factors and the psychosomatic factors, determining to what extent they influence the functional evolution in patients diagnosed with spastic quadriplegia.

## Material and methods

The research was conducted in the Pediatric Clinic of Philanthropy Municipal Hospital in Craiova, over a period of two years (January 2014 - December 2015), on a group made up of 23 children, diagnosed with spastic quadriplegia, being aged between 1 year and a half and 12 years. The study received the hospital’s ethics committee agreement and all the legal guardians of the children included in the study signed a written agreement of participation.

Patients were assessed at baseline (T1), after one year (T2) and at the study’s endpoint (T3). Throughout the study, patients received a comprehensive rehabilitation program for the correction of the motor and sensory deficits, which included hygienic-dietary measures, medication, orthopedics interventions and surgical orthotics and prosthetics, physiotherapy, physical therapy and occupational therapy [**2**].

Initially, a comprehensive evaluation (etiopathogenic, clinical and functional), that started from a thorough medical history of children (older ones) was conducted, together with a complete physical exam, and parents were chosen to identify all the risk factors.

Joint and muscle balance were conducted at each assessment. The motor function was assessed by analyzing the motor deficit, distribution, and intensity of spasticity, presence/ absence of synkinesis. Also the way each of the children could conduct active movements and take an initiative in carrying out various activities were examined, while assessing the conservation of attitude perseverance in motion, to predict motion. The clinical examination was completed by assessing sensory abilities (hearing, vision, peripheral sensitivity, discrimination, and stereognosis) and speech.

The global functional capacity evaluation included the assessment of the children’s: bed mobility, transfers and postures including the ability to crouch in the supine position, maintain positions, the ability to rise from sitting and maintain an upright posture.

The gross motor function classification system (GMFCS) was used to assess the motor function. GMFCS is very useful in the assessment of patients with spastic quadriplegia [**6**,**7**]. It analyzes active independent movements, particularly the ability to sit upright (truncal control) and to walk. Dividing the classification system into 5 degrees is based on clinically meaningful differences between the acquired motor skills.

The upper limb ability classification manual (MACS) was used to assess functionality. MACS is a reliable method for classifying manual ability [**8**,**9**]. MACS classifies the way children with cerebral palsy use their hands to manipulate objects in their daily activities. The items a child handles should be taken into consideration in light of the correlation with the child’s age. The system classifies the child’s global ability to manipulate objects, not the functionality of each hand separately.

A statistical analysis was performed by using Microsoft Excel (Microsoft Corp., Redmond, WA, USA), together with the XLSTAT add-on for MS Excel (Addinsoft SARL, Paris, France) and IBM SPSS Statistics 20.0 (IBM Corporation, Armonk, NY, USA) for data processing. The nonparametric Spearman’s rank correlation test was also used to measure the strength of association between the two ranked variables. 

## Results

A total of 23 children aged between 1 year and a half and 12 years, with a sex ratio boys: girls of 1:1.8 were included in the study. 18 children had an urban residence and 5 children came from rural areas. The demographics of patients in the study can be found in **Table 1**.

**Table 1:** Demographics of the studied group

Male	Female
8 (34.78%)	15 (65.22%)
Urban	Rural
18 (78.26%)	5 (21.74%)

From the thorough patient history, many risk factors were identified and classified according to the timeline in prenatal factors, perinatal and postnatal.

**Fig. 1:**
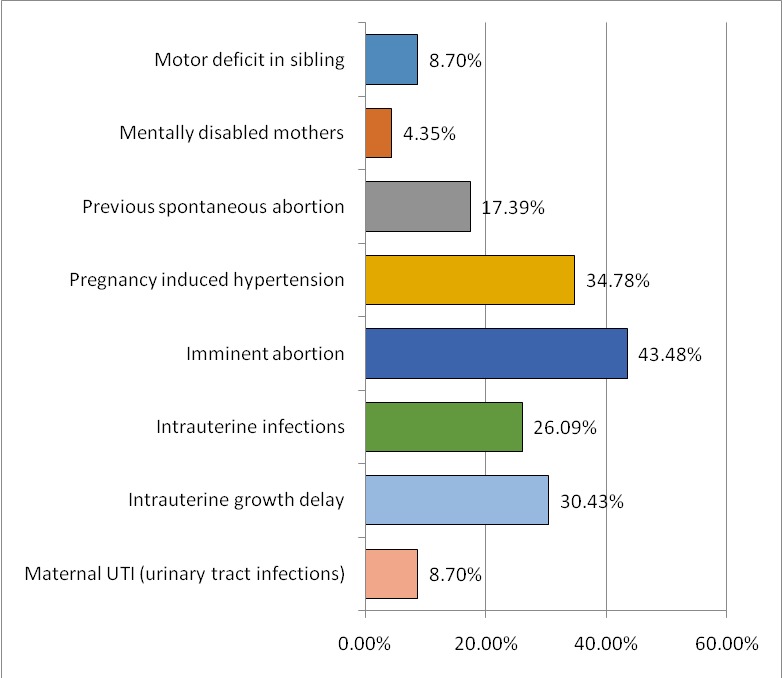
Prenatal risk factors

**Fig. 2:**
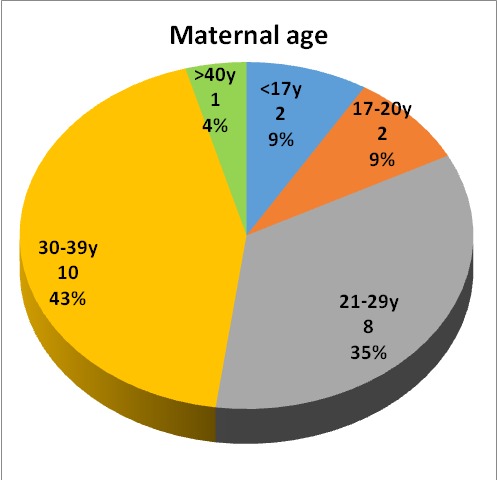
Maternal age distribution

**Fig. 3:**
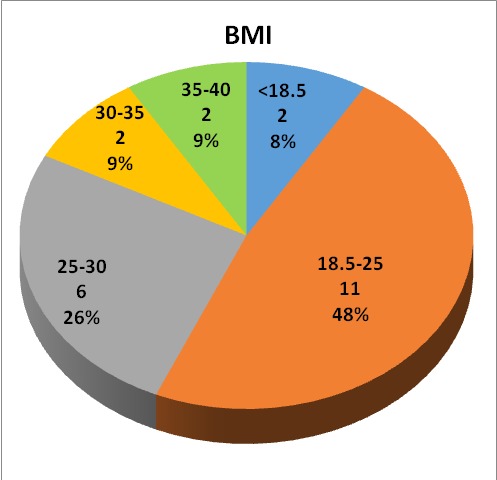
Maternal age distribution

Prenatal risk factors highlighted for children in our study, are represented in **Fig. 1**. In addition to these, age and body mass indexes of the mothers were taken into account (**Fig. 2**,**3**). Young mothers of less than 17 years or over 40 years old with a body mass index of over 25 had significant risk factors regarding the development of spastic quadriplegia.

**Fig. 4:**
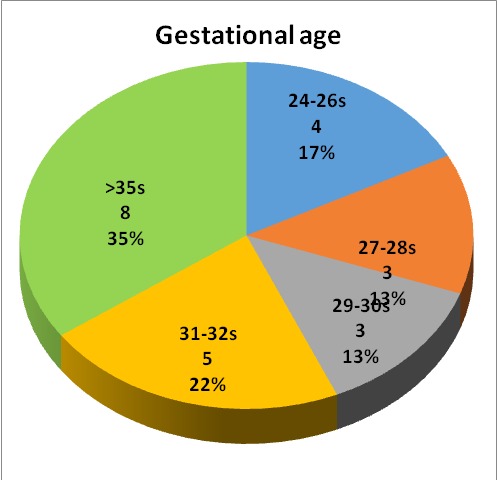
Gestational age

**Fig. 5:**
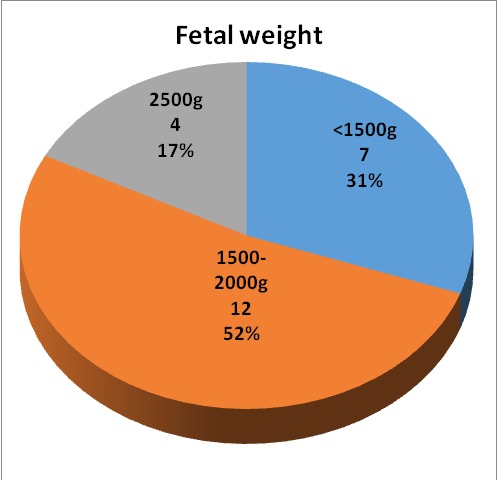
Fetal weight

**Fig. 6:**
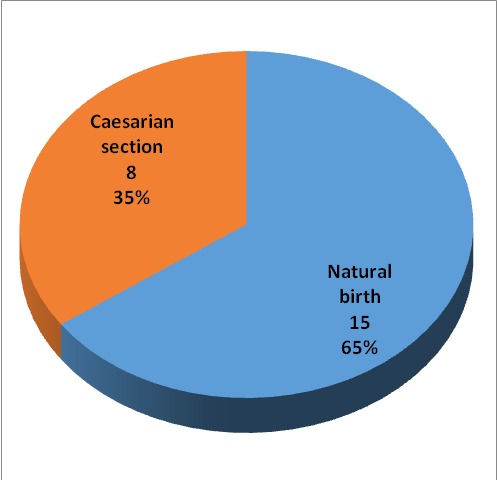
Delivery

**Fig. 7:**
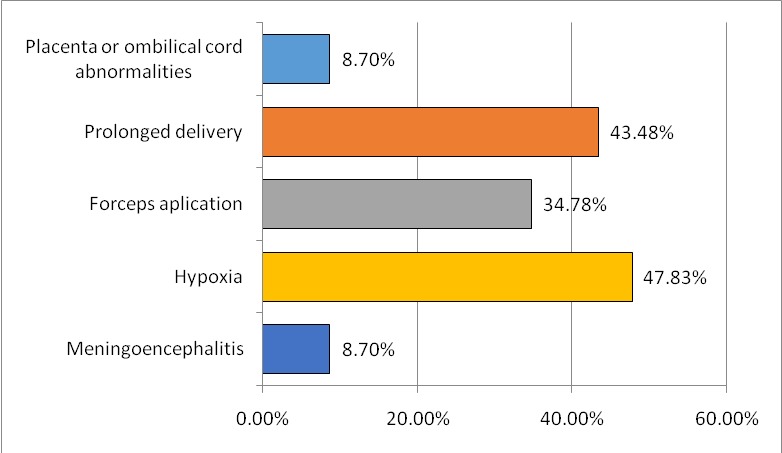
Perinatal and postnatal risk factors

The most important perinatal factors discovered were represented by a lower gestational age and a low birth weight (**Fig. 4**,**5**), and a low APGAR score. The type of delivery was also noted, natural birth or caesarean, and is illustrated in **Fig. 6**. Perinatal and postnatal factors incriminated in the appearance of spastic quadriplegia discovered in our study are illustrated in **Fig. 7**.

A direct correlation between the decrease of coarse functionality and manual ability, both initially and in dynamics, low APGAR scores, low gestational age, low birth weight and an increased BMI for the mothers were noticed. Spearman’s rho correlation coefficient (corresponding p value) and significant results between risk factors and GMFCS and MACS scores are written in bold in **Table 2**.

**Table 2:** Correlations between risk factors and GMFCS and MACS scores

	APGAR	Gestational age	Fetal weight	BMI
GMFCS T1	-0.528 (0.011)	-0.627 (0.002)	-0.502 (0.016)	0.481 (0.021)
GMFCS T2	-0.493 (0.018)	-0.579 (0.004)	-0.426 (0.044)	0.442 (0.036)
GMFCS T3	-0.457 (0.030)	-0.558 (0.006)	-0.507 (0.015)	0.372 (0.081)
MACS T1	-0.523 (0.011)	-0.639 (0.001)	-0.491 (0.019)	0.482 (0.021)
MACS T2	-0.417 (0.049)	-0.496 (0.017)	-0.417 (0.049)	0.245 (0.257)
MACS T3	-0.517 (0.012)	-0.585 (0.004)	-0.363 (0.089)	0.431 (0.041)

A statistically significant correlation between the differences in gross motor function scores and the manual ability, between the first and third evaluation was noticed, the Spearman rho correlation coefficient being 0.860 (p <0.05). A direct link between the gross motor function and the manual ability was noticed (**Table 3**). Patients with a better GMFCS score observed a significant improvement in the MACS score.

**Table 3:** Correlations between GMFCS and MACS scores

Parameter 1	Parameter 2	rho Spearman	p value
GMFCS T1	MACS T1	0.999	<0.0001
GMFCS T2	MACS T2	0.883	<0.0002
GMFCS T3	MACS T3	0.860	<0.0003

## Discussions

Cerebral palsy is the most common cause of developing neuro-motor disability in children and yet, quite often, the triggering cause remains unknown [**10**]. The prevalence of cerebral palsy is considered to be 2- 2.5 cases/ 1,000 children, sometimes reaching up to 4-6 cases/ 1000 children [**11**-**13**]. The spastic form of cerebral palsy occurs in 70-80% percent versus other forms [**14**].

The increased incidence of cerebral palsy and spastic quadriplegia is due to the fact that in the last decade there has been an improvement in postnatal care of premature babies and those with a very low birth weight [**15**]. Although perinatal care was significantly improved, most studies failed to demonstrate a significant decrease in the prevalence of cerebral palsy [**16**-**18**].

Quadriplegia is the most severe spastic cerebral palsy, characterized by severe mental retardation and bipyramidal syndrome (hypertonia with spasticity, live tendon reflexes, and pathological pyramidal-Babinski, occurring bilaterally).

Patients with spastic quadriplegia often associate movement disorders, sensory disorders and others [**19**,**20**]. Between 25 and 80% of the children diagnosed with spastic quadriplegia present comorbidities, such as sensory disturbance, malnutrition, gastrointestinal disorders, and seizures [**21**]. This condition is a very important social and economic problem, since the affected children are co-dependent.

The prevalence of spastic quadriplegia is higher in the underdeveloped and developing countries. Quadriplegia diagnosis involves various methods such as clinical and laboratory investigations, thorough psychological testing [**2**].

Through a thorough anamnesis of the patients in our study, many risk factors were identified, being classified according to the timeline in prenatal factors, perinatal and postnatal. The most important risk factors incriminated in the appearance of spastic quadriplegia in our patients were the prenatal and perinatal risk factors.

Trying to prove a direct link between the emergence of some etiologic factors and spastic quadriplegia has been the subject of numerous clinical studies [**22**-**24**]. A great variation in risk factors that can lead to spastic quadriplegia implies a detailed case history as to identify potential pre and postnatal factors that are incriminated [**25**,**26**]. Perinatal factors, such as hypoxia may promote encephalopathy and lead the further development of spastic quadriplegia. Perinatal and postnatal causes are lower as a percentage than prenatal in patients with quadriplegia [**27**].

Prenatal risk factors highlighted in our study are represented by the presence of motor deficit in a sibling, mentally disabled mothers, previous miscarriages, pregnancy-induced hypertension, imminent abortion, maternal hemorrhage, intrauterine infection, delay in intrauterine growth and maternal genitourinary infections. Apart from these, age and body mass index of mothers were taken into account. A low (under 17 years) or high (over 40 years) maternal age and a body mass index of over 25 were significant risk factors in the development of spastic quadriplegia.

Prenatal factors, including those related to the mother, have a major contribution to the development of spastic quadriplegia [**28**]. Infections during pregnancy are considered risk factors in children born at term, particularly in patients with diplegia or quadriplegia [**29**].

The occurrence of preeclampsia before 37 weeks of gestation is a true risk factor for developing cerebral palsy, largely due to a higher risk of premature birth, but also because of the influence of preeclampsia on fetal development [**30**].

Maternal obesity can be considered a risk factor in the development of spastic quadriplegia in children [**28**]. Obesity is a major public health problem worldwide [**31**,**32**].

There have been studies that have shown a direct link between the highlighting of inflammatory infiltrate from the placental level and the increased risk of spastic quadriplegia, in both preterm and term infants [**33**].

In a study, prenatal risk factors have been recognized as young age of the mother, repeated miscarriages, preeclampsia, prolonged labor, multiple births. Prenatal factors were identified as intrapartum infections, inflammatory diseases triggered during pregnancy, multiple pregnancies, and complications from previous births [**15**]. In another study, in 25% of the children diagnosed with spastic quadriplegia, the prenatal risk factors identified were malnutrition, maternal trauma, infections during pregnancy and multiple births [**34**]. The most frequent factors were represented by maternal infections during pregnancy and fever.

The most important perinatal risk factors discovered in the studied group were represented by a lower gestational age, low birth weight, and a low APGAR score. The way mothers delivered was also noted: natural or caesarean section; it did not seem to have any influence in the development of spastic quadriplegia. Other factors incriminated in the occurrence of perinatal spastic quadriplegia discovered in our study were the placental or umbilical cord abnormalities, prolonged labor, application of forceps, hypoxia.

The perinatal factors that have been shown to determine cerebral palsy and spastic quadriplegia are premature birth, low APGAR score and low birth weight. However, it was identified that up to 65% of the children diagnosed with cerebral palsy are born at term [**35**].

There was a direct link between the way birth occurred - natural or caesarean section, bleeding during pregnancy and gestational diabetes and spastic quadriplegia appearance. On the other hand, hypoxia, the newborn’s weight of less than 2,500 grams, and a gestational age between 26 and 32 weeks were correlated with the occurrence of cerebral palsy. Lack of antenatal care was also considered a risk factor in the occurrence of cerebral palsy, which imperils the life of the mother and the child [**36**,**37**].

Neonatal factors such as a gestational age below 32 weeks, weight at birth below 2,500 grams, poor intrauterine development, and maternal trauma are frequently incriminated in the development of spastic quadriplegia [**2**]. The association between a low birth weight and the appearance of spastic quadriplegia has also been demonstrated in clinical trials [**38**]. Prematurity and low birth weight represent risk factors that have been distinguished in many children diagnosed with spastic quadriplegia [**39**]. Intrapartum hypoxia is another risk factor in the occurrence of cerebral palsy and spastic quadriplegia implicitly and so is encephalopathy [**15**].

The main risk factors that have been incriminated in the appearance of spastic quadriplegia by other authors were represented by multiple pregnancies, intrapartum infection, prematurity, low birth weight, genetic factors [**25**].

Only in two children of the studied group, the development of postnatal spastic quadriplegia was caused by meningoencephalitis. Some studies have revealed that 70-80% are prenatal factors, factors related to birth are around 6%, and 10-20% of the causes of the occurrence of spastic quadriplegia are postnatal, such as bacterial meningitis, viral encephalitis, hyperbilirubinemia, trauma [**2**].

A direct correlation between the decrease of coarse functionality and manual ability was noticed, both initially and in dynamics, together with low APGAR scores, low gestational age, low birth weight, and an increased maternal body mass index.

The main causes leading to spastic quadriplegia are the prematurity (birth before 37 weeks of gestation) and antenatal and intrapartum acute fetal distress translated by a low APGAR score. In fact, these phenomena are often combined. The weight and premature birth is lower (less than 1500 grams), and lower APGAR score, the brain injuries are more common [**2**,**35**,**39**]. Maternal obesity is associated with increased rates of occurrence of gestational diabetes, preeclampsia, increasing the number of cesareans, encephalopathy, and the number of perinatal infections [**40**,**41**].

A statistically significant correlation between the gross motor functioning scores differences and the manual ability between the first and third evaluation was found, the Spearman rho correlation coefficient being 0.860 (p <0.05).

Comprehensive rehabilitation programs applied in patients with spastic quadriplegia have demonstrated a long-term efficacy by improving the functionality and quality of life in these patients [**2**,**42**].

GMFCS and MACS are very useful in assessing the functional status in patients with cerebral palsy or in those with spastic quadriplegia [**43**-**45**]. MACS and GMFCS systems are easy to apply, practical, simple and present accurate classification functionality in patients with spastic quadriplegia [**46**]. GMFCS and MACS are used as predictive factors for the evolution of functionality in patients with spastic quadriplegia [**47**,**48**].

Using GMFCS has proven a role both in clinical practice and in clinical trials due to the efficiency and accuracy used to evaluate the functional status of patients with spastic quadriplegia, leading to a more precise tracking of the utility of therapeutic options for these patients. The GMFCS classification was useful for framing motor disability in patients with spastic quadriplegia, aged 1 to 12 years. GMFCS is used to evaluate the effects of physical-kinetic therapy in patients with spastic quadriplegia [**49**-**52**].

A direct link was noticed between the gross motor function and the manual ability. A significant improvement in the MACS score, especially in younger children was seen in patients with a better GMFCS score. In a study, statistically significant correlations were determined between the gross motor function state and the manual ability of patients diagnosed with spastic quadriplegia [**53**].

By the age of 9, the motor performance begins to decline or remains the same, despite therapeutic measures in patients with a low GMFCS score - score IV and V respectively [**54**]. There was a decrease in GMFCS stage in adolescents diagnosed with spastic quadriplegia, and of the goals to be met from them to regain functionality the following can be noted: increased range of motion, increased muscle strength, and endurance, decreased spasticity, pain reduction may occur spontaneously or during movement, adequate nutrition. To achieve these objectives and to foresee these circumstances, the application of a comprehensive rehabilitation program has demonstrated its efficacy [**55**].

Other studies have shown no link between the reduction of the gross motor function and the increase in age for patients with spastic quadriplegia [**56**].

## Conclusion

In patients with a better coarse functionality score, a significant improvement in manual and scoring ability was observed. A direct link was noticed between the reduction of functionality and the coarse hand ability, both initially and in dynamics, low APGAR scores, low gestational age, low birth weight and a higher body mass index of the mother. The most important risk factors incriminated in the appearance of spastic quadriplegia were represented by the prenatal and perinatal risk factors.

**Conflict of interest**

The authors declare that they have no conflict of interest.

**Contribution note**

All the authors have contributed equally to this work.
